# Wear of composite ceramics in mixed‐material combinations in total hip replacement under adverse edge loading conditions

**DOI:** 10.1002/jbm.b.33671

**Published:** 2016-04-08

**Authors:** Mazen Al‐Hajjar, Silvia Carbone, Louise M. Jennings, Sabine Begand, Thomas Oberbach, Daniel Delfosse, John Fisher

**Affiliations:** ^1^ Institute of Medical and Biological Engineering, School of Mechanical Engineering University of Leeds Leeds UK; ^2^ Mathys Orthopädie GmbH Moersdorf Germany; ^3^ Mathys AG Bettlach Bettlach Switzerland

**Keywords:** ceramic on ceramic, hip replacement, stripe wear, microseparation, edge loading

## Abstract

Ceramic composites have performed very well under adverse edge loading conditions when used in like‐on‐like configurations, where the femoral head and acetabular cup are of the same material. The aim of this study was to determine the wear of pure alumina (Al_2_O_3_), alumina toughened zirconia (ATZ) and zirconia toughened alumina (ZTA) when used in mixed bearing combinations, under edge loading conditions due to translational mal‐positioning. The head‐on‐cup configurations of three ceramic materials were ATZ‐on‐ZTA, ZTA‐on‐ATZ, Al_2_O_3_‐on‐ATZ, ATZ‐on‐Al_2_O_3_, Al_2_O_3_‐on‐ZTA, and ZTA‐on‐Al_2_O_3_. They were tested on the Leeds II hip simulator under microseparation conditions. The bedding in and steady state wear rates of ATZ‐on‐ZTA were 1.16mm^3^/million cycles and 0.18mm^3^/million, respectively, and for ATZ‐on‐Al_2_O_3_ were 0.66 mm^3^/million cycles and 0.20 mm^3^/million, respectively. The wear rates of the other bearing combinations under these adverse microseparation conditions, Al_2_O_3_‐on‐ATZ, Al_2_O_3_‐on‐ZTA, ZTA‐on‐ATZ and ZTA‐on‐Al_2_O_3_ were very low with no clear bedding in and steady state phases, and with steady state wear rates lower than 0.11 mm^3^/million. The mixed material combinations tested in this study have shown slightly higher wear rates when compared to ATZ in like‐on‐like configuration reported previously, but superior wear resistance when compared to alumina‐on‐alumina bearings tested previously under the same adverse microseparation conditions. © 2016 The Authors Journal of Biomedical Materials Research Part B: Applied Biomaterials Published by Wiley Periodicals, Inc. J Biomed Mater Res Part B: Appl Biomater, 105B: 1361–1368, 2017.

## INTRODUCTION

The wear of ceramic‐on‐ceramic materials *in vitro* under standard hip simulator conditions is very low^1^, ^2^ (<0.1 mm^3^/million cycles) and measurement techniques cannot distinguish between the tribological performance of different current ceramic materials. However, cases of higher wear rates have been reported on early generations of alumina retrievals.^3^ Testing ceramic‐on‐ceramic bearings under standard *in vitro* simulator conditions, where perfect surgical alignments of the femoral head and the acetabular cup are assumed, did not replicate the wear mechanisms seen on some retrievals. These higher wear rates were associated with stripe wear on the femoral head with a corresponding wear area at the rim of the acetabular cup; which showed evidence of edge loading.^3^

Edge loading, where some or all of the contact area between the head and the cup lies on the rim of the acetabular cup, can occur due to rotational or translational mal‐positioning.^4^ Rotational mal‐positioning is associated with the orientation of the acetabular cup and occurs when the contact area between the femoral head and the acetabular cup intersects with the rim or edge of the acetabular cup. Translational mal‐positioning is more complex and can occur due to several clinical situations and is associated with a mismatch between the centre of the acetabular cup and the centre of the femoral head.

After the discoveries of stripe wear features on the ceramic femoral heads,^3^ Nevelos et al.^5^ have replicated edge loading on the laboratory hip simulator due to both rotational and translational mal‐positioning. Edge loading due to rotational mal‐positioning did not replicate the stripe wear mechanism seen on retrievals. Edge loading due to translational mal‐positioning was also achieved in the lab by applying microseparation conditions. Microseparation conditions, where the centres of rotation of the head and the cup were mismatched, were shown to increase the wear of ceramic‐on‐ceramic and reproduce stripe‐like wear area and the bimodal wear debris distribution similar to that seen on retrievals.^3^, ^5^, ^6^, ^7^, ^8^ This method also replicated femoral head fracture observed clinically on pure zirconia ceramic‐on‐ceramic bearings.^9^

Ceramic‐on‐ceramic bearings using modern materials such as the alumina matrix composite (AMC, BIOLOX^®^ Delta, Ceramtec) and the alumina toughened zirconia (ATZ, Ceramys^®^, Mathys Medical) had very low wear under these adverse microseparation simulator conditions, with wear rates reported below 0.15 mm^3^/million cycles for BIOLOX^®^ Delta^10^ and below 0.10 mm^3^/million cycles for Ceramys^®^,^11^ compared to the HIPed alumina ceramics^1^ (BIOLOX^®^ forte) which had a wear rate of 1.84 mm^3^/million cycles using the same conditions. Recent studies have shown high 10‐year survivorship of ceramic‐on‐ceramic bearings ranging from 95.8 to 100%^12^, ^13^, ^14^ with fracture of ceramic bearings associated with earlier generation ceramic materials.

Revision of ceramic‐on‐ceramic bearings are often associated with dislocation, impingement, loosening of the socket or fracture of either the liner or the femoral head and are not usually related to osteolysis due to the inert nature of the ceramic wear debris.^15^ Revision strategies are different depending on each individual situation. More likely both bearing surfaces will be replaced but there are cases where only the head or the cup are intact and damage free, so only the damaged surface is replaced.^15^ Modern ceramic bearings have performed very well under adverse conditions when used in like‐on‐like configurations, where the femoral head and acetabular cup are of the same material,^1^, ^10^, ^11^, ^16^ however, revision surgeries might lead to the use of different materials for the head and cup from the same manufacturer.

This study investigated the wear performance and the phase transformation of different ceramic materials with mixed head‐cup material combinations from the same manufacturer under adverse edge loading conditions on a hip joint simulator *in vitro*.

## MATERIALS AND METHODS

Six different combinations of ceramic materials, where the materials of the heads and cups were of different constituents (Table 1), were tested in this study (Mathys Orthopädie GmbH, Morsdorf, Germany). The head‐on‐cup configurations were alumina toughened zirconia‐on‐zirconia toughened alumina (ATZ‐on‐ZTA), zirconia toughened alumina‐on‐ alumina toughened zirconia (ZTA‐on‐ATZ), alumina BIONIT^®^‐on‐alumina toughened zirconia (Al_2_O_3_‐on‐ATZ), alumina toughened zirconia‐on‐alumina BIONIT^®^ (ATZ‐on‐Al_2_O_3_),alumina BIONIT^®^‐on‐zirconia toughened alumina (Al_2_O_3_‐on‐ZTA) and zirconia toughened alumina‐on‐ alumina BIONIT^®^ (ZTA‐on‐Al_2_O_3_) with diametrical clearance between 70 and 90 µm.

**Table 1:** Constituent of the Different Materials Used in this Study

	Al_2_O_3_	ZrO_2_
Alumina BIONIT^®^ (Al_2_O_3_)	100%	–
Alumina toughened zirconia (ATZ)	20%	80%
Zirconia toughened alumina (ZTA)	75%	25%

**Figure 1:**
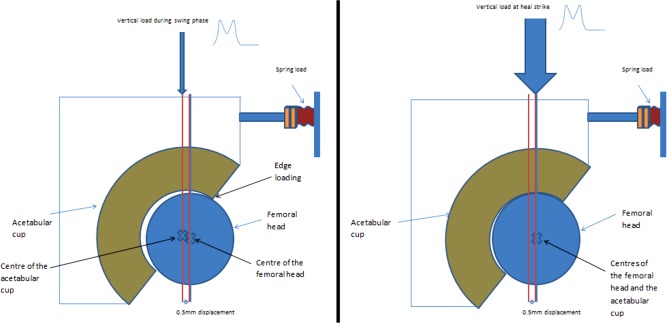
Schematics showing the positions of the femoral heads and acetabular cups during dynamic microseparation conditions.

The Leeds II Physiological Anatomical Hip Joint Simulator was used to test the six bearing combinations (*n* = 3) under edge loading conditions for four million cycles. All femoral heads used had a diameter of 28 mm. Edge loading conditions were achieved by introducing microseparation^5^ (translational mal‐position) conditions to the standard gait cycle. The gait cycle comprised of flexion/extension (−15°/+30°), internal/external rotation (±10°), and a twin peak load of a maximum of 3 kN. Microseparation was achieved by lateralising the head relative to the acetabular cup by 0.4–0.5 mm during swing phase and edge loading occurred at heel strike (Figure 1).

The acetabular cups were taper locked into metallic shells which were mounted at an inclination angle equivalent to an *in vivo* cup inclination angle of 55°. The femoral heads were taper locked onto femoral stems which were mounted in the simulator using PMMA acrylic resin. New‐born calf serum (25%, v/v) was used as a lubricant which was supplemented with 0.03% of sodium azide to inhibit bacterial growth. The lubricant was changed every one third of a million cycles. Wear was assessed gravimetrically; the components were weighed using a Mettler AT201 balance (0.01 mg readability) before and after the test and at every million cycles. One way ANOVA and *post hoc* Least significance difference (LSD) test was performed for statistical analysis and significance levels were taken at *p* < 0.05.

Surface roughness over the wear stripe generated due to edge loading was assessed using two‐dimensional contacting profilometry (Form Talysurf series, Taylor Hobson, UK). Approximately 15 mm long traces were taken post test over the wear stripes and analyzed by applying a Gaussian filter and the recommended cutoff according to ISO 4288‐1997. The roughness values over the wear areas were compared to the roughness values of the unworn surface determined pretest for each material separately and a Student's *t test* was used with significance taken at *p* < 0.05.

The femoral heads were inspected using a scanning electron microscope to visualise the wear scars/stripes in comparison to the unworn regions. Further, the monoclinic fraction of ZrO_2_ was measured on the surface of the femoral heads with X‐ray‐diffraction following Rietveld refinement. It is sufficient to analyze the fraction of monoclinic ZrO_2_ because this is the crucial phase with respect to the mechanical properties and the phase stability in a hydrothermal environment or an *in‐vivo* environment. The low temperature degradation (LTD) of zirconia or zirconia containing materials leads to an increase of the undesirable monoclinic phase content. So by determining the fraction of the monoclinic phase, the stability of the ceramic materials can be described. The XRD Analysis was performed using a diffractometer XRD3003 TT (GE Sensing Inspection Technology) with Bragg–Brentano geometry and Cu–K‐alpha radiation. The diffractometer was also equipped with a goebel mirror for parallel beam and a scintillation detector. During the measurements the focus of the X rays was directly within the worn area. For comparison, reference measurements were carried out on the unworn area for each individual head and a Student's *t* test was carried out with significance taken at *p* < 0.05. The phase composition work was done in collaboration with the Fraunhofer Institut for Ceramic Technologies and Systems, Germany.

**Figure 2:**
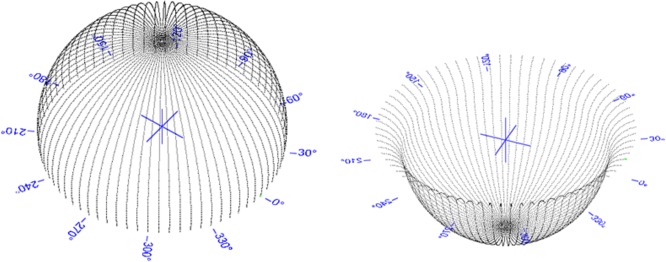
Data points taken by the CMM on the surfaces of the femoral heads and acetabular cups.

Three‐dimensional reconstructions of the surface of the femoral heads and acetabular cups were obtained by using a coordinate measuring machine (CMM, Legex 322, Mitutoyo, Japan) and SR3D software (Tribology solutions, UK). The femoral heads and acetabular cups were measured by taking 72 traces over each surface with 5° spacing about the vertical axis (Figure 2). Each trace started at the pole of the component and had a 0.2 mm pitch resulting in a total number of 9,936 points on the femoral heads and 8928 points on the acetabular cups. In order to get the best resolution out of the CMM machine, for these particular measurements, a 3 mm stylus was used with a vertical probe set up. SR3D software (Tribosol, UK) was used to visualize the size, shape and penetration depth of the wear. One‐way ANOVA and *post hoc* least significance difference (LSD) test was performed for statistical analysis and significance levels were taken at *p* < 0.05.

## RESULTS

**Figure 3:**
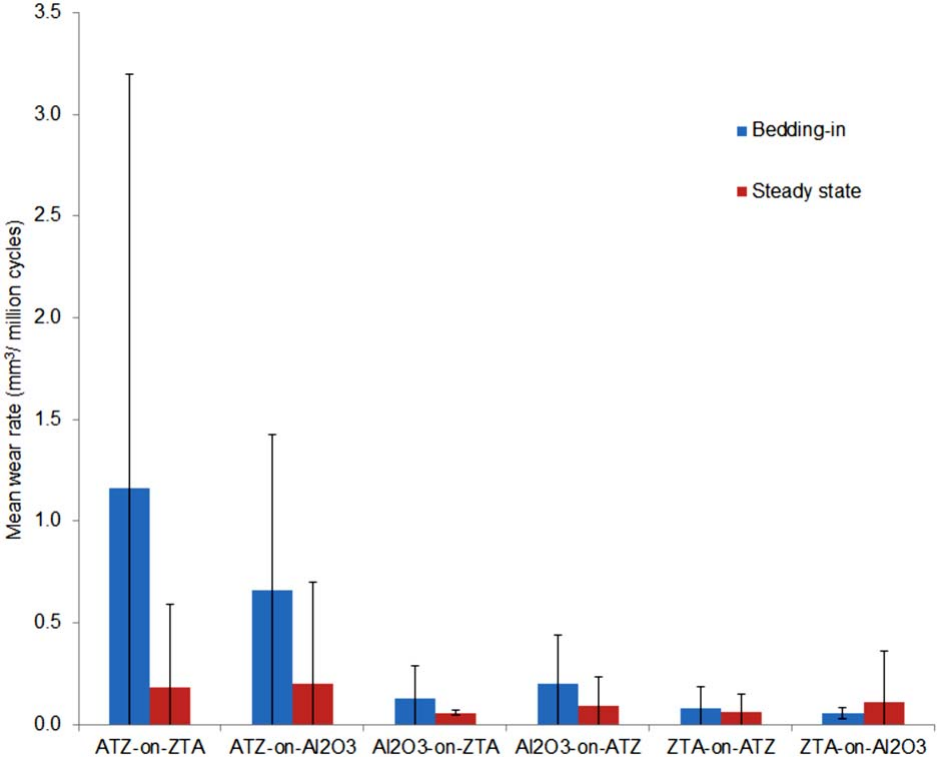
Mean wear rates of all bearings combinations tested under adverse microseparation conditions. The bedding in wear is between zero and one million cycles and steady state between one and four million cycles. Error bars represent 95% confidence limit. The only significant differences were found between the bedding in wear rate of ATZ‐on‐ZTA and the bedding in wear rates of Al_2_O_3_‐on‐ATZ, Al_2_O_3_‐on‐ZTA, ZTA‐on‐ATZ and ZTA‐on‐Al_2_O_3_ (*p* = 0.004, *p* = 0.007, *p* = 0.003, *p* = 0.003, respectively).

The wear of ATZ‐on‐ZTA and ATZ‐on‐Al_2_O_3_ was biphasic with a bedding‐in wear rate between zero and one million cycles of testing under microseparation conditions and a lower steady state wear rate between one and four million cycles of testing (Figure 3). The bedding in and steady state wear rates of ATZ‐on‐ZTA were 1.16 mm^3^/million cycles and 0.18 mm^3^/million, respectively and those of ATZ‐on‐Al_2_O_3_ were 0.66 mm^3^/million cycles and 0.20 mm^3^/million, respectively (Figure 3). The wear rates of the other bearings combinations under these adverse microseparation conditions, Al_2_O_3_‐on‐ATZ, Al_2_O_3_‐on‐ZTA, ZTA‐on‐ATZ, and ZTA‐on‐Al_2_O_3_, were very low with no clear difference between the bedding in and steady state phases (Figure 3). However, for comparison, the wear rates of all combinations were split into bedding in phase between 0 and 1 million cycles and steady state phase between 1 and 4 million cycles. The steady state wear rates of Al_2_O_3_‐on‐ATZ, Al_2_O_3_‐on‐ZTA, ZTA‐on‐ATZ, and ZTA‐on‐Al_2_O_3_ over the four million cycles of test under adverse microseparation conditions were all lower than 0.11 mm^3^/million cycles (Figure 3).

The bedding in wear rate of ATZ‐on‐ZTA was not significantly different (*p* = 0.11) than that of ATZ‐on‐Al_2_O_3_, but was significantly higher (*p* < 0.05) than the bedding in wear rate of Al_2_O_3_‐on‐ATZ, Al_2_O_3_‐on‐ZTA, ZTA‐on‐ATZ, and ZTA‐on‐Al_2_O_3_. There was no significant difference (*p* = 0.46) in the steady state wear rate of all combinations.

The surface roughness generally increased over the wear stripe area on the femoral head and the wear area at the rim of the acetabular cups when compared to unworn regions (Table 2). The increase was significant for ATZ and Al_2_O_3_ heads articulating in the ATZ‐on‐ZTA, ATZ‐on‐Al_2_O_3_, Al_2_O_3_‐on‐ATZ, and Al_2_O_3_‐on‐ZTA combinations (*p* = 0.05, *p* = 0.02, *p* = 0.05, *p* = 0.02, respectively) but not significant for ZTA heads articulating against ATZ or Al_2_O_3_ (*p* = 0.10 and *p* = 0.36, respectively). ZTA and Al_2_O_3_ cups showed no significant (*p* = 0.36) increase in Ra values when articulated against ATZ heads however the surface roughness over the wear area significantly increase (*p* = 0.002, *p* < 0.001, *p* < 0.001, and *p* = 0.04, respectively) for all the cups in Al_2_O_3_‐on‐ATZ, Al_2_O_3_‐on‐ZTA, ZTA‐on‐ATZ, and ZTA‐on‐Al_2_O_3_ combinations. Before testing under microseparation conditions, the roughness Ra values were below 0.010 µm for all femoral heads and acetabular cups of all materials.

**Figure 4:**
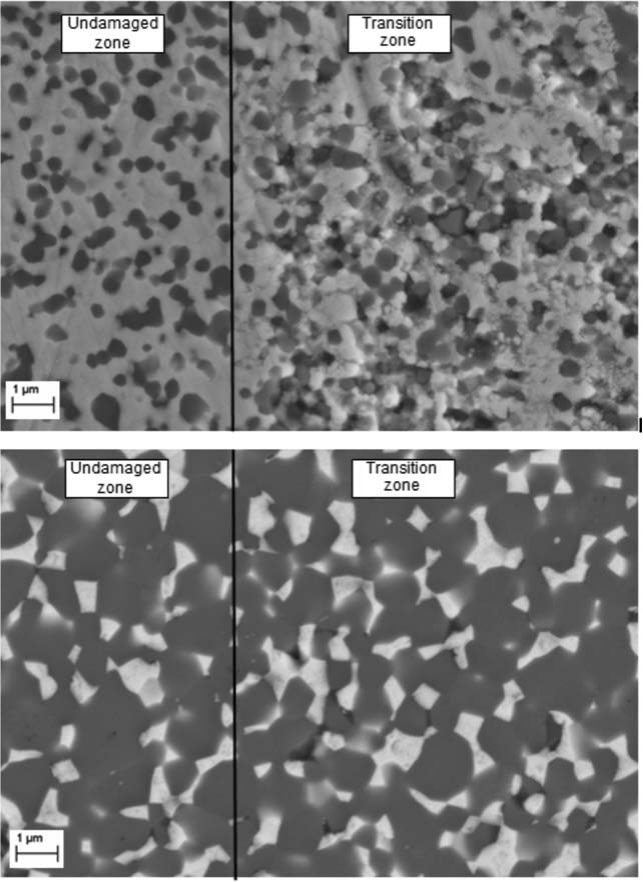
SEM images taken over the edge of the wear stripe area on the ATZ femoral head articulation against Al_2_O_3_ liner (top) and the ZTA femoral head articulation against ATZ liner (bottom).

The stripe wear region of the Al_2_O_3_ femoral heads was characterised by a markedly visual transition zone indicated by local material fatigue of the alumina grain. Over this area, considerable material fatigue occurred followed by individual grain pull‐outs. The surface of the ZTA ceramic head was less affected by the edge loading conditions with less grain break‐outs apparent (Figure 4). For the ATZ femoral heads, the surface was characterized by single grain‐pull‐outs, and small regions of material fatigue were visible (Figure 4).

**Table 2:** Roughness *R_a_* (µm) Parameter Over the Wear Area of the Femoral Head and Acetabular Cup After Testing Under Severe Microseparation Condition for Four Million Cycles

		ATZ‐on‐ZTA	ATZ‐on‐Al_2_O_3_	Al_2_O_3_‐on‐ATZ	Al_2_O_3_‐on‐ZTA	ZTA‐on‐ATZ	ZTA‐on‐ Al_2_O_3_
Heads	Mean	0.022	0.016	0.023	0.045	0.006	0.014
	95% confidence limit	0.014	0.005	0.032	0.025	0.002	0.027
Cups	Mean	0.008	0.014	0.024	0.037	0.032	0.025
	95% confidence limit	0.003	0.015	0.012	0.010	0.010	0.034

The results of the X‐ray‐analysis in terms of phase content of the monoclinic zirconia of the heads with standard deviation (as calculated by the software of the X‐ray diffraction machine) are detailed in Table 3. In the original state, the phase content of monoclinic zirconia was lower for the ZTA heads since the lower zirconia content in the ZTA ceramic meant there was also less monoclinic zirconia. The measured monoclinic zirconia content in the worn ‘stripe’ regions of ZTA heads was slightly higher following microseparation simulation conditions but the increase was not significant for any of the heads tested (*p* = 0.1 for ATZ‐on‐ZTA, *p* = 0.07 for ATZ‐on‐Al_2_O_3_, and *p* = 0.3 for ZTA‐on‐ATZ). ATZ heads exhibited, in the original state, a typical monoclinic zirconia content of about 3 wt %. Independent of the articulating counterpart a slightly higher monoclinic content was observed but this increase could be estimated as noncritical. On all measuring points the values were consistent with the International Standard ISO 13356, which specifies a requirement of <20% of the monoclinic zirconia content for pure Y‐TZP.

**Table 3:** Mean Phase Content of Monoclinic Zirconia (wt%) with Standard Deviation on the Femoral Head Following Microseparation Conditions

	ATZ‐on‐ZTA		ATZ‐on‐Al_2_O_3_		ZTA‐on‐ATZ	
	Unworn	Worn	Unworn	Worn	Unworn	Worn
Monoclinic zirconia (wt%)	3.40 ± 1.6	5.8 ± 1.6	3.2 ± 1.5	6.3 ± 1.6	0.6 ± 0.5	0.8 ± 0.5

**Figure 5:**
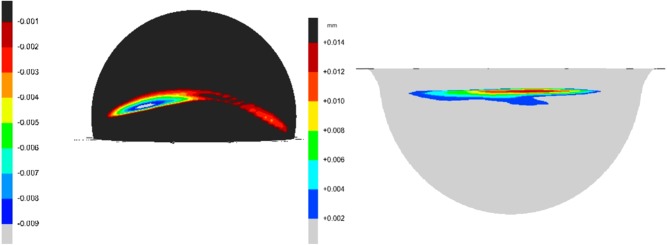
Three**‐**dimensional representation of the wear stripe on the femoral head and the wear area at the rim of the acetabular cup measured using the Legex 322 CMM (Mitutoyo, Japan) and analysed using SR3D software (TriboSol, UK).

**Figure 6:**
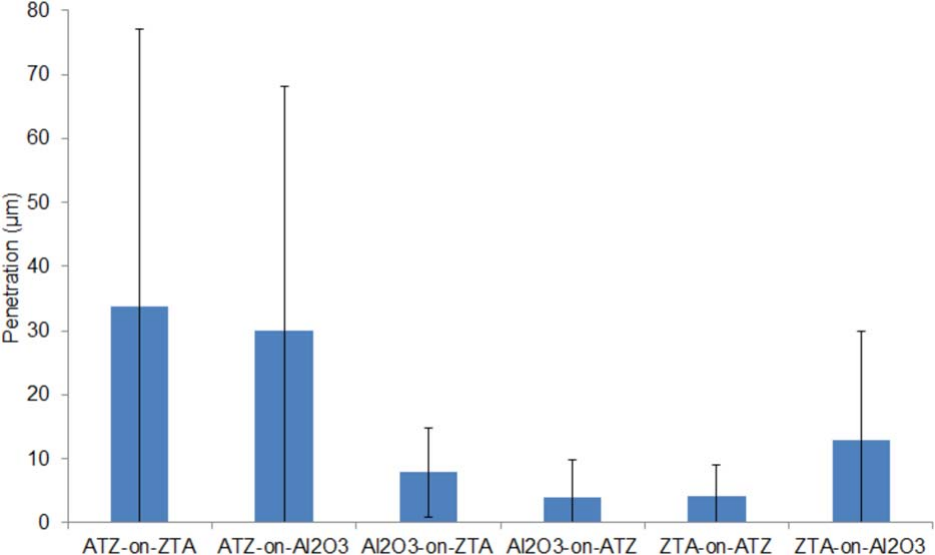
The mean maximum penetration depth over the wear stripe on the femoral heads measured on the CMM after testing under severe microseparation conditions for four million cycles. Error bars represent 95% confidence limit. The significant differences were found between the ATZ femoral heads from the ATZ‐on‐ZTA, and ATZ‐on‐ Al_2_O_3_ combinations and the ATZ, ZTA and Al_2_O_3_ heads from the mixed Al_2_O_3_‐on‐ATZ (*p* = 0.008 and *p* = 0.019, respectively), Al_2_O_3_‐on‐ZTA (*p* = 0.003 and *p* = 0.008, respectively), and ZTA‐on‐ATZ (*p* = 0.003 and *p* = 0.008, respectively). The penetration depth on ZTA head from the ZTA‐on‐Al_2_O_3_ combination was significantly lower than that of the ATZ heads from ATZ‐on‐ZTA but not from the ATZ‐on‐Al_2_O_3_ combination (*p* = 0.025 and *p* = 0.059, respectively) bearings.

Stripe wear was observed on all the femoral heads after testing under edge loading conditions due to microseparation conditions with a corresponding wear area at the rim of the acetabular cups (Figure 5). The penetration depths over the ATZ femoral heads were 34 and 30 µm for the ATZ‐on‐ZTA and ATZ‐on‐Al_2_O_3_ combinations, respectively, with no significant difference between the two groups (*p* = 0.65) (Figure 6). The ATZ, ZTA, and Al_2_O_3_ heads from the mixed Al_2_O_3_‐on‐ATZ, Al_2_O_3_‐on‐ZTA, ZTA‐on‐ATZ and ZTA‐on‐Al_2_O_3_ bearings showed mean penetration depth of 13 µm or lower after four million cycles of test under microseparation conditions (Figure 6). There were significant differences in penetration depths between the ATZ femoral heads from the ATZ‐on‐ZTA, and ATZ‐on‐ Al_2_O_3_ combinations and the ATZ, ZTA and Al_2_O_3_ heads from the mixed Al_2_O_3_‐on‐ATZ (*p* = 0.008 and *p* = 0.019, respectively), Al_2_O_3_‐on‐ZTA (*p* = 0.003 and *p* = 0.008, respectively), and ZTA‐on‐ATZ (*p* = 0.003 and *p* = 0.008, respectively). The penetration depth on ZTA head from the ZTA‐on‐Al_2_O_3_ combination was significantly lower than that of the ATZ heads from ATZ‐on‐ZTA but not from the ATZ‐on‐Al_2_O_3_ combination (*p* = 0.025 and *p* = 0.059, respectively) bearings.

## DISCUSSION

Ceramic‐on‐ceramic bearings can potentially meet the demands of young and active patients. This is highlighted by its low wear properties^1^, ^2^, ^10^, ^11^, ^16^, ^17^, ^18^ and bioinert wear debris.^19^ Improvements in material constituency, toughness and design of ceramic bearings have made them tribologically superior to previous generations of ceramic materials,^10^, ^11^, ^16^, ^20^ and allowed the development of larger bearing sizes, which can technically provide improved range of motion. Also, current issues with other bearing materials such as the osteolytic potential of polyethylene wear debris^21^ and the tissue reactions to metal wear debris^22^ have made modern ceramic‐on‐ceramic bearings an attractive alternative.

Retrievals studies have changed our understanding of preclinical testing as some of the wear mechanisms observed on ceramic‐on‐ceramic and metal‐on‐metal bearings did not match wear mechanisms obtained under the standard walking simulator conditions.^3^, ^23^, ^24^, ^25^ Retrieval studies have shown evidence of stripe wear and edge loading.^5^ Edge loading occurs when the wear patch or contact area between the head and the cup intersect with the acetabular rim due to either rotational or translational mal‐positioning.^4^ Rotational mal‐positioning is related to acetabular cup inclination and version angles whereas translational mal‐positioning is defined as a mismatch between the centres of rotation of the cup and the head. Translational mal‐positioning could occur due to many clinical reasons such as head offset deficiency, medialized cup, stem subsidence, impingement, subluxation and laxity of surrounding soft tissue. *In vitro* studies have shown that the wear of metal‐on‐metal bearings is sensitive to both rotational and translational mal‐positioning.^26^, ^27^, ^28^, ^29^ However, only edge loading due to translational mal‐positioning replicated stripe wear and clinically relevant wear rates and wear debris of ceramic‐on‐ceramic bearings.^5^, ^30^ This study investigated the wear of ceramic materials, pure alumina, alumina toughened zirconia and zirconia toughened alumina, when used in mixed‐bearing combinations under clinically relevant adverse *in vitro* conditions.

In this study, only mixed bearing combinations were studied with no control group however, a previous study using the same methodology used in this study demonstrated the high wear resistance of the alumina‐toughened‐zirconia and the zirconia‐toughened‐alumina under adverse edge loading conditions when used in a like‐on‐like (ATZ‐on‐ATZ and ZTA‐on‐ZTA) configuration. The wear rates reported were below 0.1 mm^3^/million cycles compared to a steady state wear rate of 0.55 mm^3^/million cycles for Al_2_O_3_‐on‐Al_2_O_3_. In this study, all mixed bearing combinations, ATZ‐on‐ZTA, ATZ‐on‐Al_2_O_3_, Al_2_O_3_‐on‐ATZ, Al_2_O_3_‐on‐ZTA, ZTA‐on‐ATZ, and ZTA‐on‐Al_2_O_3_ had steady state wear rates lower than that of Al_2_O_3_‐on‐ Al_2_O_3_ (<0.20 mm^3^/million cycles).

Al_2_O_3_‐on‐ATZ, Al_2_O_3_‐on‐ZTA, ZTA‐on‐ATZ, and ZTA‐on‐Al_2_O_3_ bearing combinations showed very low wear rates with no sign of bedding‐in wear rate. The lack of the initial relatively higher bedding in wear rate of these bearings using composite materials shows a huge tribological advantage compared to Al_2_O_3_‐on‐Al_2_O_3_, as *in vivo* bedding in wear occurs under different conditions where the contact area between the head and the cup changes. The absence of the bedding in wear rate will reduce the overall wear over the duration of implant use *in vivo*. The penetration depths on the heads were higher for ATZ articulating against ZTA or Al_2_O_3_ cups than Al_2_O_3_ heads articulating against ATZ or ZTA cups and ZTA head articulating against ATZ and Al_2_O_3_ cups, which could be due to the lower stiffness and hardness of ATZ material compared to alumina and ZTA materials. The hardness of ATZ material (16.4 GPa) is lower than that of ZTA (17.9 GPa) and Al_2_O_3_ (18.2 GPa) giving rise to a relatively softer on a harder material combination, leading to increased wear compared to a harder on softer material such as ZTA‐on‐ATZ. This observation is consistent with the metal‐on‐polyethylene and ceramic‐on‐metal combinations where the wear is lower when the femoral head is the harder material. The Young's modulus of ATZ material (261 GPa) is significantly lower of that of ZTA (363 GPa) and alumina material (392 GPa) (internal report, Mathys Orthopädie GmbH, Moersdorf, Germany).

The penetration depths obtained in this study were comparable to the penetration depths of Al_2_O_3_ heads articulating in like‐on‐like configuration^11^ and significantly lower than the penetration depths measured on BIOLOX^®^ forte femoral heads (90  µm)^16^ tested under the same simulator conditions.

Surface characterization indicated a small increase to the surface roughness over the wear area on the femoral head. The wear area formed on the femoral head was stripe‐like with a corresponding wear area near the rim of the acetabular cup. The stripe wear formed on all femoral heads was shallower than the stripe of wear formed on Al_2_O_3_ heads articulating against Al_2_O_3_ cups.^11^ This shows the higher wear resistance of the new bearing combinations compared to Al_2_O_3_‐on‐Al_2_O_3_ bearings under the adverse edge loading conditions.

Although the wear rates of Al_2_O_3_, ATZ, and ZTA in mixed bearings combinations were slightly higher than in like‐on‐like configuration,^11^ the wear rates obtained in this study in any configuration were very low. To compare to other bearings materials, the wear rate of metal‐on‐metal bearings has been reported to be in the range of 2–9 mm^3^/million cycles under microseparation conditions.^27^, ^31^, ^32^

Referring to the XRD‐measurements it can be suggested that on all the tested femoral heads, an increase of monoclinic zirconia content was observed. Comparing all tested mixed combinations with like‐on‐like combinations, the like‐on‐like pairing^11^ ATZ‐on‐ATZ offered the best resistance in terms of tetragonal/monoclinic zirconia transformation under edge loading conditions due to translational mal‐positioning. ATZ offers a huge advantage over pure alumina and pure zirconia ceramic materials. It combined the advantages of the high toughness of zirconia and stability of alumina material. ATZ material has a fracture toughness of 7.9 MPa m^1/2^ compared to 3.6 MPa m^1/2^ for alumina. Previous studies have shown the instability of pure zirconia materials under edge loading due to phase transformation in the crystal structure leading to fracture of femoral heads.^9^ Although an increase in the monoclinic zirconia content was observed in this study, the level of increase was still within acceptable levels.

## CONCLUSION

Ceramic‐on‐ceramic materials using modern ceramic materials are becoming more attractive due to the superior tribological performance under adverse clinically relevant *in vitro* conditions. The mixed material combinations (ATZ‐on‐ZTA, ATZ‐on‐Al_2_O_3_, and Al_2_O_3_‐on‐ATZ, Al_2_O_3_‐on‐ZTA and ZTA‐on‐ Al_2_O_3_) tested in this study have shown slightly higher wear rates when compared to previously tested ATZ in like‐on‐like configuration,^11^ but superior wear resistance when compared to alumina Al_2_O_3_‐on‐Al_2_O_3_ bearings tested previously under the same adverse microseparation conditions.^1^, ^11^ In this study, the lowest wearing mixed material combination was ZTA‐on‐ATZ which had low wear rate similar to that of ATZ‐on‐ATZ.
